# The effectiveness of surgical vs conservative interventions on pain and function in patients with shoulder impingement syndrome. A systematic review and meta-analysis

**DOI:** 10.1371/journal.pone.0216961

**Published:** 2019-05-29

**Authors:** Goris Nazari, Joy C. MacDermid, Dianne Bryant, George S. Athwal

**Affiliations:** 1 School of Physical Therapy, Faculty of Health Science, Western University, London ON Canada; 2 Collaborative Program in Musculoskeletal Health Research, Bone and Joint Institute, Western University, London ON Canada; 3 Roth McFarlane Hand and Upper Limb Centre, St. Joseph’s Hospital, London, ON Canada; University of Mississippi Medical Center, UNITED STATES

## Abstract

**Objective:**

To assess the effectiveness of surgical vs conservative interventions on pain and function in patients with subacromial impingement syndrome.

**Design:**

Systematic review and meta-analysis of randomized controlled trials.

**Setting:**

Clinical setting.

**Participants:**

Patients 18 years and older with subacromial impingement syndrome.

**Intervention/Comparison:**

Surgical intervention plus postoperative physiotherapy / placebo surgery plus physiotherapy or physiotherapy only.

**Main outcome measures:**

Pain and function.

**Results:**

11 RCTs (n = 919) were included. The pooled results displayed no statistically or clinically different between surgery plus physiotherapy vs physiotherapy alone on pain levels at 3-, 6-months, 5- and 10 years follow up (moderate quality, 3 RCTs, 300 patients, WMD -0.39, 95% CI: -1.02 to 0.23, p = 0.22; moderate quality, 3 RCTs, 310 patients, WMD -0.36, 95% CI: -1.02 to 0.29, p = 0.27; low quality, 1 RCT, 109 patients, WMD -0.30, 95% CI: -1.54 to 0.94, p = 0.64; low quality, 1 RCT, 90 patients, WMD -1.00, 95% CI: -0.24 to 2.24, p = 0.11) respectively. Similarly, the pooled results were not statistically or clinically different between groups for function at 3-, 6-month and 1-year follow ups (very low quality, 2 RCTs, 184 patients, SMD 0.11, 95% CI: -0.57 to 0.79, p = 0.75; moderate quality, 3 RCTs, 310 patients, SMD 0.15, 95% CI: -0.14 to 0.43, p = 0.31; very low quality, 2 RCTs, 197 patients, SMD 0.11, 95% CI: -0.46 to 0.69, p = 0.70) respectively.

**Conclusion:**

The effects of surgery plus physiotherapy compared to physiotherapy alone on improving pain and function are too small to be clinically important at 3-, 6-months, 1-, 2-, 5- and ≥ 10-years follow up.

## Introduction

Shoulder pain is regarded as one of the most frequently reported non-traumatic complaints that arise from the arm, neck and shoulder regions [1], with high prevalence rates across multiple countries [2–5]. Prevalence rates of shoulder pain among the general population have been estimated to be approximately 11% in Canada [2], 14% in UK [3], 27% in US [4], and 22% in Australia (North West Adelaide) [5]. Shoulder pain is believed to be a significant symptom of shoulder/ subacromial impingement syndrome–a set of clinical and radiological findings that pertains to tendinitis and bursitis of the rotator cuff and adjacent tissues [1,6]. Shoulder impingement syndrome is associated with reduction in function, quality of life and mobility [7].

The available treatment options for shoulder impingement syndrome include both conservative approaches mainly exercise, and surgical techniques–arthroscopic surgical decompression. It is suggested that exercise be considered as the primary conservative treatment option for shoulder impingement [8]. The Steuri (2017) systematic review demonstrated that exercise treatment programs yield superior outcomes in pain when compared to non-exercise controls in patients with shoulder impingement (very low quality, 5 RCTs, 189 patients, SMD -0.94, 95% CI: -1.69 to -0.19) [8]. Similarly, improvements in function were superior in exercise treatment programs compared to non-exercise controls, (very low quality, 4 RCTs, 202 patients, SMD -0.57, 95% CI: -0.85 to -0.29) [8]. Alternatively, arthroscopic surgical decompression option may be indicated in patients with persistent severe subacromial shoulder pain along with functional limitations that have not improved in response to conservative treatment options [9]. The Steuri (2017) review also indicated that there was insufficient evidence to display whether exercise is as good as surgery [8].

Multiple newly published individual RCTs have shown that a surgical approach such as arthroscopic surgical decompression improves both shoulder pain and disability, while others have found similar benefits through physical therapy interventions–mainly exercises. Paavola (2018) trial displayed a statistically significant benefit of arthroscopic surgical decompression over exercise therapy in shoulder pain at rest and on arm activity at 2-years follow up [10]. Similarly, Beard (2018) trial indicated statistically significant improvements in patient-important outcomes with subacromial decompression at 1-year follow up [9]. However, these improvements were of uncertain clinically importance [9]. Conversely, Farfaras (2018) trial demonstrated that subacromial decompression yielded higher scores in patient-rated function that were clinically meaningful over physical therapy alone after a minimum of 10-years follow-up [11].

Systematic reviews (Saltychev 2015; Steuri 2017) [1,8], have provided useful, but conflicting insights on the current state of the evidence concerning the effectiveness of surgery vs conservative approaches on clinical outcome in patients with shoulder impingement syndrome. Saltychev (2015) concluded that there is moderate evidence indicating surgical treatment is no more effective than active exercises on reducing pain intensity caused by shoulder impingement [1], whereas, Steuri (2017) concluded that there was insufficient evidence to display whether exercise is as good as surgery [8]. Given the increase in the number of newly published randomized controlled trials (n = 6) on this topic, an up-to-date review which incorporates the most recently available evidence is needed. Therefore, the objective of this review was to quantify the effects of surgical vs conservative interventions on clinical outcomes of pain and function in patients with subacromial impingement syndrome at 3- and 6-months, 1-, 2-, 5- and ≥ 10- years follow up.

## Methods

We followed the Preferred Reporting Items for Systematic Reviews and Meta-Analyses (PRISMA) and Cochrane collaboration guidelines [12–13]. (S1 PRISMA Checklist)

PROSPERO registration number: CRD 42018115632.

### Eligibility criteria

Studies were included in this systematic review if the below criteria were met [1,8]:

- *Design*: randomized controlled trial (RCT), published in a peer reviewed journal,
- *Participants*: patients 18 years and older with subacromial pain/impingement syndrome,
- *Intervention vs Comparison*: trials that compared patients who received surgical intervention and postoperative rehabilitation vs rehabilitation only, and vs placebo surgical intervention and postoperative rehabilitation
- *Outcomes*: pain and function

Studies that included patients with rotator cuff tears, degenerative arthritis, rheumatoid arthritis of glenohumeral joint, adhesive capsulitis/ shoulder fractures / previous surgery, that were conference abstracts or posters were excluded from this systematic review.

### Information sources

We conducted systematic electronic searches to identify relevant randomized controlled trials in MEDLINE, EMBASE, CINAHL and PubMed from January 1998 to November 2018. Several different combinations of keywords were used, such as: “shoulder impingement”, “subacromial impingement syndrome”, “randomized controlled trials”, “arthroscopic subacromial decompression”, “open subacromial decompression”, “rehabilitation”, “conservative”, “physiotherapy” (S1 File). In addition, we also performed a search in the PROSPERO database and carried out a manual search of the reference lists of the previous systematic reviews and the references of all the included articles.

### Study selection

Selection of individual RCTs involved two independent reviewers (GN and JM) who carried out the systematic electronic searches in each database. Duplicate studies were identified and removed. Next, we independently screened the titles and abstracts. We then retrieved in full text any study marked include or uncertain by either reviewer. Finally, we carried out an independent full text review to determine final eligibility.

### Data collection process

Two independent researchers (GN and JM) extracted the data from the eligible trials. Data extraction included the authors, year, country, study population, sample size, age, intervention/comparison group, primary and secondary outcomes, follow up periods and the protocols for the surgical interventions and postoperative rehabilitation. When insufficient data were presented, (GN) contacted the authors by email and requested further data.

### Assessment of risk of bias in individual studies

Two independent review authors (GN and JM) assessed the trials for risk of bias. The risk of bias assessment was carried out using the Cochrane Risk of Bias tool [12]. The Cochrane Risk of Bias tool is based on 7 items, random sequence generation, allocation concealment, blinding of participants and personnel, blinding of outcome assessment, incomplete outcome data, selective reporting and other bias [12]. We defined the other bias category as trials that did not include statements on sources of funding. We then summarized the assessment of risk of bias per outcome across trials as provided in the Cochrane Handbook for Systematic Reviews of Interventions, as Low risk of bias (if low risk of bias was judged for random sequence generation, allocation concealment, blinding of participants/personnel, blinding of outcome assessment, incomplete outcome data, selective reporting and other bias); as Unclear risk of bias (if unclear risk of bias was judged for one or more of random sequence generation, allocation concealment, blinding of participants/personnel, blinding of outcome assessment, incomplete outcome data, selective reporting and other bias); and, as High risk of bias (if high risk of bias was judged for one or more of random sequence generation, allocation concealment, blinding of participants/personnel, blinding of outcome assessment, incomplete outcome data, selective reporting and other bias) [12]. (S1 Appendix)

### Assessing the quality of evidence

The GRADE approach for systematic reviews was used to assess the quality of evidence related to each outcome and to summarize the extent of our confidence in the estimates of the effect [14–20]. The GRADE approach considers the risk of bias, publication bias, consistency of findings (, precision, and the applicability of the overall body of literature to provide a rating of quality of evidence (high, moderate, low, or very low) per outcome [14–20].

### Summary measures

To quantify and interpret our data, a Minimally Clinically Important Differences (MCID) of 1.5 points (0–10) for pain [21]. Furthermore, a standard deviation of 0.5 points for function and pain (if a scale other than 0–10 was used, for example PainDETECT) were used to interpret meaningful change [22]. Timing of outcome assessments were reported at 3- and 6-months, 1-, 2-, 5- and ≥ 10-years follow up. A standard deviation of 0.5 points for function was used due to the fact that the MCID thresholds of the outcome measures used in the included RCTs were not yet established. In addition, various RCTs utilized different outcome measures to quantify function, therefore, considering this paucity of the reported MCID thresholds and an attempt to facilitate meta-analysis of the data from the included RCTs, a standard deviation of 0.5 points for function was used based on Norman et al. (2004) proposed approach [22].

### Subgroup analysis and exploring heterogeneity

In the presence of heterogeneity (inconsistency), we planned to conduct the following subgroup analyses (a priori): trials at low risk of bias (low risk of bias in allocation concealment and blinding of outcome assessor if objective outcomes were used) would show a smaller effect size and postoperative rehabilitation received. An I^2^ estimate of at least 50% and a statistically significant Chi^2^ statistic (P = 0.10) was interpreted as evidence of a substantial problem with heterogeneity [23].

### Synthesis of results

We performed 19 meta-analyses of trials comparing surgical intervention and postoperative rehabilitation vs rehabilitation only, and vs placebo surgical intervention and postoperative rehabilitation, at 3- and 6-months, 1-, 2-, 5- and ≥ 10-years follow up. We used the Review Manager 5.3 (RevMan 5.3) software to conduct our review and a random-effects model to pool outcomes. For outcomes of the same construct that were measured using a different metric, we used the standardized mean difference (SMD). If all eligible trials measured an outcome using the same metric, we used a weighted mean difference (WMD).

## Results

### Study selection

**Fig 1:**
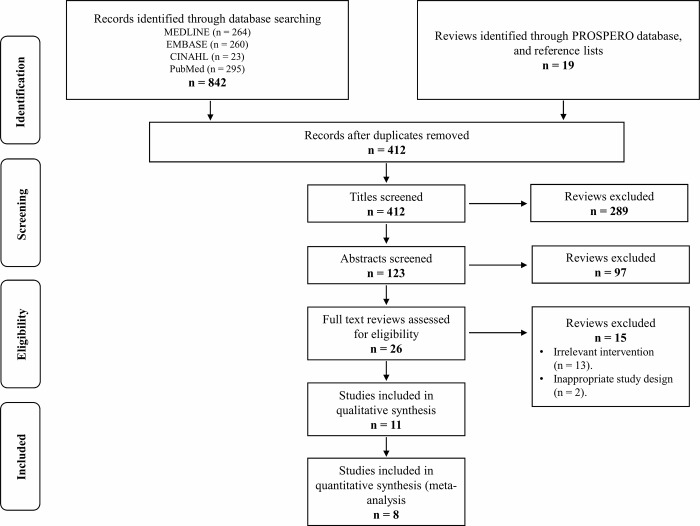
Selection of studies for inclusion in the systematic review.

Initially, our search yielded 861 publications. After removal of the duplicates, 412 articles remained and were screened using their title and abstract; leaving 26 articles selected for full text review. Of these, 11 RCTs were eligible [9–11, 24–31]. The flow of studies through the selection process is presented in Fig 1.

### Study characteristics

The 11 eligible RCTs were conducted between 1998 and 2018 and included 919 patients (376 surgery plus physiotherapy, 273 physiotherapy alone, and 166 placebo surgery plus physiotherapy, 104 no treatment) [9–11, 24–31]. Study size ranged from 39 to 313 patients. Trials were conducted in Norway, Sweden, Denmark, Finland and United Kingdom [9–11, 24–31]. A summary description of all the included RCTs is displayed in Table 1.

**Table 1:** Summary of included randomized controlled trials.

Study	Country	Population	Groups	Outcomes	Follow ups	Surgical Interventions	Conservative/No Interventions
Brox 1999 [24]	Norway	Patients with rotator cuff disease for at least three months	Surgery + Ex. n = 45 (29 men, 16 women), Age 48.0 years Exercise n = 50 (22 men, 28 women), Age 47.0 years	-Pain -Function	3, 6 months & 2.5 years.	Arthroscopic surgery (bursectomy and resection of the anterior and lateral part of the acromion and the coracoacromial ligament). Postoperative rehabilitation was started on the first postoperative day. Physiotherapy was started within the first week. The exercises prescribed by the surgeon were performed against low resistance and repeated many times. Patients visited a physiotherapist where they lived, so several physiotherapists were engaged, and somewhat different approaches used. Unrestricted activities were usually allowed after four to six weeks.	To eliminate gravitational forces and to start the exercises the arm was suspended in a sling fixed to the roof. Relaxed repetitive movements (first rotation, then flexion—extension, and finally abduction-adduction) were performed for about an hour in a daily training session. Patients were supervised twice weekly. On the other days they followed the same exercise programme at home. Resistance was added gradually to strengthen the short shoulder rotator and the scapular stabilising muscles. The training continued for three to six months, with the supervision gradually being reduced.
Rahme 1998 [25]	Sweden	Patients with subacromial impingement syndrome	Surgery + physiotherapy n = 21 Physiotherapy n = 18 (19 males, 23 females), age 42.0 years	-Pain	6 months & 1-year.	Open anterior acromioplasty according to Neer. Attention was paid to the portion of the acromion that may extend beyond the anterior border of the clavicle. Followed by physiotherapy.	Information on functional anatomy/ biomechanics, advice on how to avoid wear and tear positions, unload movements of the shoulder, normalize scapulohumeral rhythm, postural awareness, strengthening of the shoulder muscles and endurance training.
Haahr 2005; 2006 [26–27]	Denmark	Patients with subacromial impingement	Surgery + Physiotherapy n = 41 (12 males, 29 females), Age 44.3 years Physiotherapy n = 43 (14 males, 29 females) Age 44.5 years	-Pain -Function	3, 6 months, 1 year, & 4–8 years.	The treatment consisted of bursectomy with partial resection of the antero-inferior part of the acromion and the coracoacromial ligament. Two experienced surgeons undertook all procedures and recorded their findings on a predetermined proforma. Before discharge, the patient was instructed in performing light movements of the arm within the limits of pain. Stitches were removed by general practitioners after 10 days. At the same time, the patient was instructed by a physiotherapist to carry out increasingly active exercises, including exercises for strengthening the rotator cuff muscles	The treatments started with application of heat, cold packs, or soft tissue treatments. This was followed by active training of the periscapular muscles (rhomboid, serratus, trapezoid, levator scapulae, and pectoralis minor muscles) and strengthening of the stabilising muscles of the shoulder joint (the rotator cuff). This was done within the limits of pain. During the first two weeks the patient was seen three times weekly, during the next three weeks twice weekly, and during the last seven weeks once weekly. The patients were encouraged to continue to do active exercises at home on a daily basis. After carrying out the full programme for at least 12 weeks, the patients were encouraged to continue the programme two to three times a week.
Ketola 2009; 2013; 2017 [28–29,31]	Finland	Patients with shoulder impingement syndrome	Surgery + Exercise n = 70 (29 men, 41 women) Age 46.4 years Exercise n = 70 (23 men, 47 women) Age 47.8 years	-Pain -Disability	1, 2, 5 & 10 years	Arthroscopic decompressions. An interscalenic or supraclavicular brachial plexus block was applied for regional anaesthesia. Bony landmarks were palpated and marked. Glenohumeral stability and passive range of movement were tested. The arthroscope was introduced through a standard posterior portal and a systematic recording of the articular cartilage, labrum and ligaments, biceps tendon, and the intra-articular rotator cuff was performed. The same standard portal was used to reach the subacromial space. Debridement and decompression were done through an anterolateral portal by shaver and / or vaporiser. If the coracoacromial ligament felt tight or thick, it was released. Acromioplasty was then performed, starting anteriorly and progressing posterolaterally with a burr drill. The range of movement was tested under arthroscopic visualisation to check for any local impingement, plus, similar exercises as the other group. NSAIDs was allowed as needed. Subacromial corticosteroid injections were permitted.	Information was first given by a trained physiotherapist. A home programme was individually planned for each patient according to the same principles. The aim was to restore painless and normal mobility of the shoulder complex and to increase the dynamic stability of the glenohumeral joint (supra- and infraspinatus, teres minor, and subscapular muscles) and the scapula (trapezoid, rhomboid, serratus anterior, and pectoralis minor muscles).29 Elasticated stretch bands and light weights were used in training, which was based on long painless series and repetitions aiming at tendon strengthening. The sessions were performed at least four times a week using nine different exercises with 30 to 40 repetitions three times. As the self-assessed ability and strength improved, resistance was increased, and repetitions diminished. NSAIDs was allowed as needed. Subacromial corticosteroid injections were permitted.
Farfaras 2014; 2018 [11,30]	Sweden	Patients with subacromial impingement syndrome	Open acromioplasty + Physiotherapy n = 15 (7 males, 8 females) age 52.4 years Arthroscopic acromioplasty + Physiotherapy n = 19 (7 males, 12 females) age 48.9 years. Physiotherapy n = 21 (13 males, 8 females) age 49.9 years	-Function	31 months (~2.5 years) & Min. 10 years (range 10–17 years)	Open acromioplasty was performed according to Rockwood and Lyons with the patient in the beach chair position. The procedure started with an anterior, lateral 5-cm skin incision. The deltoid muscle was split and detached from the anterior third of the acromion and the acromioclavicular joint capsule. After exposing the anterior edge of the acromion, the tendinous anterior third of the acromion was elevated dorsally prior to removing bone. This manoeuvre exposed the coracoacromial ligament. An osteotome was used to remove the anterior edge and the lateral portion of the undersurface of the acromion. The removed bone included the attachment of the coracoacromial ligament. The piece of bone was about 6–9 mm wide and 20 mm long. Proximal to the coracoid, the coracoacromial ligament was cut. Palpation of the undersurface of the acromion was performed to detect any fragments of bone or prominences. The undersurface of the acromioclavicular joint was palpated and inspected. If osteophytes were present, they were excised. No acromioclavicular joint resections were performed. Finally, the medial flap of the deltoid was sutured to the capsule of the acromioclavicular joint, and the lateral flap was sutured to the origin of the deltoid before closure of the wound. Arthroscopic acromioplasty was performed according to Ellman with the patient in the lateral decubitus position. A traction device was applied to the arm, and a tension to the arm corresponding to 40 N was applied. The shoulder was in 10° of flexion and 40° of abduction. The bony landmarks of the shoulder (the acromion, the clavicle, the acromioclavicular joint, the coracoid and the coracoacromial ligament) were marked with a pen. A portal for the arthroscope was created on the dorsal side of the shoulder. The gleno-humeral joint was first evaluated for cartilage changes, disorder of the biceps tendon, labrum and the rotator cuff. Using the same arthroscopic portal, the subacromial space was visualised and a bursectomy was performed with a shaver introduced from a lateral portal. A resection of the anterior edge of the acromion of about 5–8 mm was then carried out, followed by a resection of about 5–8 mm of the anterior–inferior third of the undersurface of the acromion all the way to the acromioclavicular joint.	Physiotherapy group received treatments according to the method described by Böhmer. The purpose of the treatment is to let the patients find their normal kinematics of the shoulder, without experiencing pain. The gravitational forces on the arm were removed by suspending the arm in a sling fixed to the ceiling. The training programme started with rotational movements of the arm. As soon as the patient was able to perform these motions without pain, flexion/extension movements were added, followed by abduction/adduction exercises. The training programme postulates everyday practice of at least 60 min. The load was gradually increased in order to strengthen the rotator cuff and the scapula-stabilising muscles. In the final stage of the programme, the patients replaced some exercises with corresponding leisure activities. The programme was performed twice a week under the supervision of a physiotherapist and the rest of the days at home for a period of three to six months. In order to secure similar treatment, all the patients were trained at five local physiotherapy centres by physiotherapists using the same standardised protocol.
Paavola 2018[10]	Finland	Patient with shoulder impingement syndrome	Arthroscopic subacromial decompression + post-operative care including exercise n = 59 (17 males, 42 females) Age 50.5 years Diagnostic arthroscopy (placebo surgery) + post-operative care including exercise n = 63 (17 males, 46 females), Age 50.8 years Exercise Therapy n = 71 (24 males, 47 females) Age 50.4 years	-Pain -Function	3,6 months, 1 & 2 years	Arthroscopic subacromial decompression procedures involved the debridement of the entire subacromial bursa and resection of the bony spurs and the projecting anterolateral undersurface of the acromion, was carried out with a shaver, burr, and / or electrocoagulation. Post-operative care consisted of one visit to an independent physiotherapist, blind to the group assignment, for guidance and instructions for home exercises. Diagnostic arthroscopy involved examination of the glenohumeral joint and subacromial space with the use of standard posterior and lateral portals and a 4 mm arthroscope with the patient under general anaesthesia, usually supplemented with an interscalene brachial plexus block. We did an intraarticular and subacromial assessment of the rotator cuff integrity.	Exercise therapy–Supervised, progressive, individually designed physiotherapy was started within two weeks of randomisation, using a standardised protocol that relied primarily on daily home exercises as well as 15 visits to an independent physiotherapist
Beard 2018 [9]	United Kingdom	Patients with subacromial pain	Arthroscopic subacromial decompression + physiotherapy n = 106 (52 males, 54 females), Age 52.9 years Investigational arthroscopy (placebo surgery) + physiotherapy n = 103 (51 males, 52 females), Age 53.7 years No treatment n = 104 (52 males, 52 females), Age 53.2 years	-Function -Pain	6 and 12 months	Arthroscopic subacromial decompression was done according to routine practice under general anaesthetic. It involved removal of bursa and soft tissue within the subacromial space, release of the coraco-acromial ligament, and removal of the subacromial bone spur through posterior and lateral portals. Investigational arthroscopy (placebo surgery) was also done under general anaesthetic through a posterior portal. Patients underwent routine investigational arthroscopy of the joint, rotator cuff tendons, and subacromial bursa, with the operation done in exactly the same manner as decompression. A lateral skin incision was made to simulate a lateral portal, but no instruments were introduced through this incision. The intervention did not involve surgical removal of any bone, bursal tissue, other soft tissue or release of the coracoacromial ligament. The procedure involved inspection and irrigation of the glenohumeral joint (arthroscopy) and the subacromial bursa (bursoscopy).	No treatment (monitoring) involved patients attending one reassessment appointment with a specialist shoulder clinician, 3 months after entering the study but with no planned intervention. The patients in the no-treatment group had no prescribed physiotherapy or steroid injections.

### Risk of bias assessment in the individual studies

**Fig 2:**
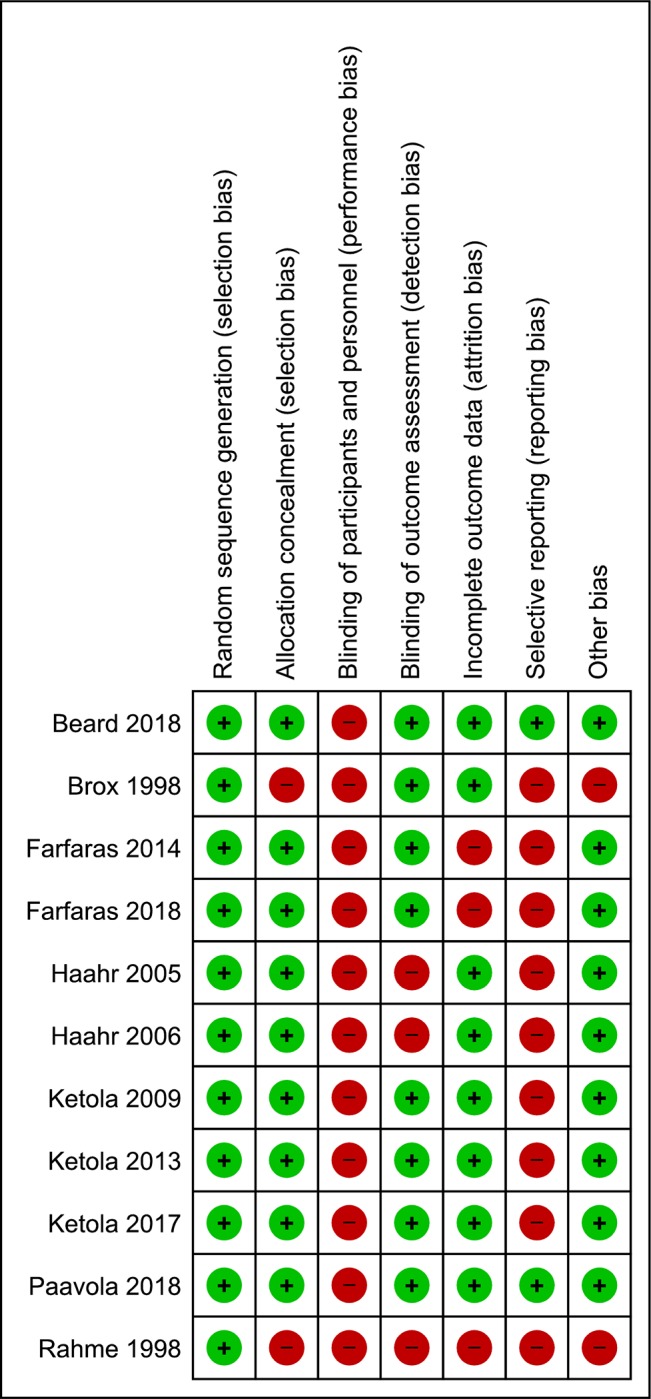
Risk of bias summary: Review authors’ judgements about each risk of bias item for each included study.

The risk of bias assessment is presented in Fig 2. Performance bias (lack of or inadequate blinding of participants who could influence how interventions, including co-interventions are performed/administered) was rated at high risk in all the included trials (n = 11) [9–11, 24–31]. Selective Reporting bias were rated at high risk in nine trials [11, 24–31]. Detection bias (lack of or inadequate blinding of participants who could influence the measurement or interpretation of outcomes) and attrition bias (significant or imbalanced missing outcome data) were rated at high risk in three trials [11,25–27,30]. Selection bias and other biases (RCTs with no statements on sources of funding) were rated at high risk in two trials [24–25]. Overall, all eleven included RCTs were rated at high risk of bias [9–11, 24–31].

### GRADE Evidence Profile (EP) and Summary of Findings (SoF)

The EP (Tables 2 and 3) displays a detailed quality assessment and includes a judgment of each factor that determined the quality of evidence for each outcome. The SoF tables (Tables 4–7) include an assessment of the quality of evidence for each outcome.

**Table 2:** GRADE evidence profile: Surgery plus physiotherapy vs physiotherapy alone.

Quality Assessment						Summary of Findings			
Outcome (No. of studies; design)	Limitations	Inconsistency	Indirectness	Imprecision	Publication Bias	Surgery plus Physiotherapy	Physiotherapy alone	SMD / WMD (95% CI)	Quality
Pain at 3 months (3 RCTs) [10,26,28]	Serious limitations	No serious inconsistency	No serious indirectness	No serious imprecisions	Unlikely	138/300	162/300	WMD -0.39 (-1.02–0.23)	⊕⊕⊕⊝ Moderate
Pain at 6 months (3 RCTs) [10,26,28]	Serious limitations	No serious inconsistency	No serious indirectness	No serious imprecisions	Unlikely	144/310	166/310	WMD -0.36 (-1.02–0.29)	⊕⊕⊕⊝ Moderate
Pain at 1 year (3 RCTs) [10,26,28]	Serious limitations	No serious inconsistency	No serious indirectness	No serious imprecisions	Unlikely	147/317	170/317	WMD -0.67 (-1.23 –-0.11)	⊕⊕⊕⊝ Moderate
Pain at 2 years (2 RCTs) [10, 28]	Serious limitations	No serious inconsistency	No serious indirectness	No serious imprecisions	Unlikely	127/261	134/261	WMD -0.67 (-1.23 –-0.12)	⊕⊕⊕⊝ Moderate
Pain at 5 years (1 RCT) [29]	Serious limitations	N/A	No serious indirectness	Serious imprecisions	Unlikely	57/109	52/109	WMD -0.30 (-1.54–0.94)	⊕⊕⊝⊝ Low
Pain at 10 years (1 RCT) [31]	Serious limitations	N/A	No serious indirectness	Serious imprecisions	Unlikely	44/90	46/90	WMD 1.00 (-0.24–2.24)	⊕⊕⊝⊝ Low
Function at 3 months (2 RCTs) [26,28]	Serious limitations	Serious inconsistency	No serious indirectness	Serious imprecisions	Unlikely	84/184	100/184	SMD 0.11 (-0.57–0.79)	⊕⊝⊝⊝ Very low
Function at 6 months (3 RCTs) [26,28]	Serious limitations	No serious inconsistency	No serious indirectness	No serious imprecisions	Unlikely	144/310	166/310	SMD 0.15 (-0.14–0.43)	⊕⊕⊕⊝ Moderate
Function at 1 year (2 RCTs) [26,28]	Serious limitations	Serious inconsistency	No serious indirectness	Serious imprecisions	Unlikely	92/197	105/197	SMD 0.11 (-0.46–0.69)	⊕⊝⊝⊝ Very low
Function at 2–2.5 years (3 RCTs) [28,30]	Serious limitations	No serious inconsistency	No serious indirectness	Serious imprecisions	Unlikely	146/301	155/301	SMD 0.31 (0.08–0.54)	⊕⊕⊝⊝ Low
Function at 5 years (1 RCT) [29]	Serious limitations	N/A	No serious indirectness	Serious imprecisions	Unlikely	57/109	52/109	SMD 0.14 (-0.24–0.51)	⊕⊕⊝⊝ Low
Function at ≥10 years (2 RCTs) [11,31]	Serious limitations	No serious inconsistency	No serious indirectness	Serious imprecisions	Unlikely	62/136	74/136	SMD 0.22 (-0.12–0.56)	⊕⊕⊝⊝ Low

**Table 3:** GRADE evidence profile: Surgery plus physiotherapy vs placebo surgery plus physiotherapy.

Quality Assessment						Summary of Findings			
Outcome (No. of studies; design)	Limitations	Inconsistency	Indirectness	Imprecision	Publication Bias	Surgery plus Physiotherapy	Placebo Surgery plus Physiotherapy	SMD / WMD (95% CI)	Quality
Pain at 3 months (1 RCT) [10]	Serious limitations	N/A	No serious indirectness	Serious imprecisions	Unlikely	54/109	55/109	SMD 0.11 (-0.27–0.48)	⊕⊕⊝⊝ Low
Pain at 6 months (2 RCTs) [9,10]	Serious limitations	No serious inconsistency	No serious indirectness	No serious imprecisions	Unlikely	140/283	143/283	SMD 0.08 (-0.15–0.32)	⊕⊕⊕⊝ Moderate
Pain at 1 year (2 RCTs) [9,10]	Serious limitations	No serious inconsistency	No serious indirectness	No serious imprecisions	Unlikely	122/250	128/250	SMD 0.06 (-0.21–0.33)	⊕⊕⊕⊝ Moderate
Pain at 2 years (1 RCTs) [10]	Serious limitations	N/A	No serious indirectness	Serious imprecisions	Unlikely	59/118	59/118	SMD -0.26 (-0.62–0.10)	⊕⊕⊝⊝ Low
Function at 6 months (2 RCTs) [9,10]	Serious limitations	No serious inconsistency	No serious indirectness	Serious imprecisions	Unlikely	141/286	145/286	SMD -0.20 (-0.48–0.08)	⊕⊕⊝⊝ Low
Function at 1 year (1 RCT) [9]	Serious limitations	N/A	No serious indirectness	Serious imprecisions	Unlikely	76/157	81/157	SMD 0.07 (-0.24–0.38)	⊕⊕⊝⊝ Low
Function at 2 years (1 RCT) [10]	Serious limitations	N/A	No serious indirectness	Serious imprecisions	Unlikely	59/118	59/118	SMD 0.26 (-0.10–0.62)	⊕⊕⊝⊝ Low

**Table 4:** Summary of findings. Surgery plus physiotherapy vs physiotherapy alone (Pain).

Population: patients with subacromial impingement syndrome. Settings: inpatient clinics. Intervention: Surgery plus Physiotherapy Comparison: Physiotherapy alone Follow up: 3-, 6-months and 1-, 2-, 5- and 10-years			
Outcomes	WMD (95% C.I.)	No of participants (RCTs)	Quality of the evidence (GRADE)
**Pain (3-months):** [10,26,28] VAS (0–10) Lower values indicate improved pain.	WMD -0.39 (-1.02–0.23)	300 (3 RCTs)	⊕⊕⊕⊝ Moderate ^1^
**Pain (6-months):** [10,26,28] VAS (0–10) Lower values indicate improved pain.	WMD -0.36 (-1.02–0.29)	310 (3 RCTs)	⊕⊕⊕⊝ Moderate ^1^
**Pain (1-year):** [10,26,28] VAS (0–10) Lower values indicate improved pain.	WMD -0.67 (-1.23–-0.11)	317 (3 RCTs)	⊕⊕⊕⊝ Moderate ^1^
**Pain (2-years):** [10, 28] VAS (0–10) Lower values indicate improved pain.	WMD -0.67 (-1.23–-0.12)	261 (2 RCTs)	⊕⊕⊕⊝ Moderate ^1^
**Pain (5-years):** [29] VAS (0–10) Lower values indicate improved pain.	WMD -0.30 (-1.54–0.94)	109 (1 RCT)	⊕⊕⊝⊝ Low ^1^ ^2^
**Pain (10-years):** [31] VAS (0–10) Lower values indicate improved pain.	WMD 1.00 (-0.24–2.24)	90 (1 RCT)	⊕⊕⊝⊝ Low ^1^ ^2^

Abbreviations: VAS; visual analogue scale, MD; mean difference, CI; confidence interval.

^1^We downgraded by one level due to high risk of bias.

^2^We downgraded by one level due to a relatively small sample size.

**Table 5:** Summary of findings. Surgery plus physiotherapy vs physiotherapy alone (Function).

Population: patients with subacromial impingement syndrome. Settings: inpatient clinics. Intervention: Surgery plus Physiotherapy Comparison: Physiotherapy alone Follow up: 3-, 6-months and 1-, 2–2.5, 5- and 10–17 years			
Outcomes	SMD (95% C.I.)	No of participants (RCTs)	Quality of the evidence (GRADE)
**Function (3-months):** [26,28] Constant/Shoulder Disability Questionnaire. (0–100) Higher values indicate improved function.	SMD 0.11 (-0.57–0.79)	184 (2 RCTs)	⊕⊝⊝⊝ Very low ^1^ ^2^ ^3^
**Function (6-months):** [26,28] Constant/ Shoulder Disability Questionnaire. (0–100) Higher values indicate improved function.	SMD 0.15 (-0.14–0.43)	310 (3 RCTs)	⊕⊕⊕⊝ Moderate ^1^
**Function (1-year):** [26,28] Constant/ Shoulder Disability Questionnaire. (0–100) Higher values indicate improved function.	SMD 0.11 (-0.46–0.69)	197 (2 RCTs)	⊕⊝⊝⊝ Very low ^1^ ^2^ ^3^
**Function (2–2.5 years):** [28,30] Constant/ Shoulder Disability Questionnaire. (0–100) Higher values indicate improved function.	SMD 0.31 (0.08–0.54)	301 (3 RCTs)	⊕⊕⊝⊝ Low ^1^ ^2^
**Function (5-years):** [29] Constant/ Shoulder Disability Questionnaire. (0–100) Higher values indicate improved function.	SMD 0.14 (-0.24–0.51)	109 (1 RCT)	⊕⊕⊝⊝ Low ^1^ ^2^
**Function (≥ 10-years):** [11,31] Constant/ Shoulder Disability Questionnaire. (0–100) Higher values indicate improved function.	SMD 0.22 (-0.12–0.56)	136 (2 RCTs)	⊕⊕⊝⊝ Low ^1^ ^2^

Abbreviations: VAS; visual analogue scale, SMD; standardized mean difference, CI; confidence interval.

^1^We downgraded by one level due to high risk of bias.

^2^We downgraded by one level due to a relatively small sample size.

^3^We downgraded by one level due to inconsistency.

**Table 6:** Summary of findings. Surgery plus physiotherapy vs placebo surgery plus physiotherapy (Pain).

Population: patients with subacromial impingement syndrome. Settings: inpatient clinics. Intervention: Surgery plus Physiotherapy Comparison: Placebo Surgery plus Physiotherapy Follow up: 3-, 6-months and 1- and 2-years			
Outcomes	WMD/SMD (95% C.I.)	No of participants (RCTs)	Quality of the evidence (GRADE)
**Pain (3-months):** [10] VAS (0–10) Lower values indicate improved pain.	SMD 0.11 (-0.27–0.48)	109 (1 RCTs)	⊕⊕⊝⊝ Low ^1^ ^2^
**Pain (6-months):** [9,10] VAS/PainDETECT (0–10) Lower values indicate improved pain.	SMD 0.08 (-0.15–0.32)	283 (2 RCTs)	⊕⊕⊕⊝ Moderate ^1^
**Pain (1-year):** [9,10] VAS/PainDETECT (0–10) Lower values indicate improved pain.	SMD 0.06 (-0.21–0.33)	250 (2 RCTs)	⊕⊕⊕⊝ Moderate ^1^
**Pain (2-years):** [10] VAS (0–10) Lower values indicate improved pain.	SMD -0.26 (-0.62–0.10)	118 (1 RCT)	⊕⊕⊝⊝ Low ^1^ ^2^

Abbreviations: VAS; visual analogue scale, MD; mean difference, SMD; standardized mean difference, CI; confidence interval.

^1^We downgraded by one level due to high risk of bias.

^2^We downgraded by one level due to a relatively small sample size.

**Table 7:** Summary of findings. Surgery plus physiotherapy vs placebo surgery plus physiotherapy (Function).

Population: patients with subacromial impingement syndrome. Settings: inpatient clinics. Intervention: Surgery plus Physiotherapy Comparison: Placebo Surgery plus Physiotherapy Follow up: 6-months and 1- and 2-years			
Outcomes	SMD (95% C.I.)	No of participants (RCTs)	Quality of the evidence (GRADE)
**Function (6-months):** [9,10] Constant (0–100) Higher values indicate improved function.	SMD -0.20 (-0.48–0.08)	286 (2 RCTs)	⊕⊕⊝⊝ Low ^1^ ^2^
**Function (1-year):** [9] Constant (0–100) Higher values indicate improved function	SMD 0.07 (-0.24–0.38)	157 (1 RCT)	⊕⊕⊝⊝ Low ^1^ ^2^
**Function (2-years):** [10] Constant (0–100) Higher values indicate improved function	SMD 0.26 (-0.10–0.62)	118 (1 RCT)	⊕⊕⊝⊝ Low ^1^ ^2^

Abbreviations: VAS; visual analogue scale, SMD; standardized mean difference, CI; confidence interval.

^1^We downgraded by one level due to high risk of bias.

^2^We downgraded by one level due to a relatively small sample size.

### Participants / Outcomes

The 11 included RCTs recruited patients with subacromial impingement syndrome/subacromial pain and rotator cuff disease [9–11, 24–31]. Pain levels were measured using a Visual Analogue Scale (VAS) [10,26, 28–29, 31], and PainDETECT [9]. Function was measured using Constant [9–11, 26, 30], and Shoulder Disability Questionnaire[28–29,31]. The follow-up period was up to 17 years postoperatively.

### Surgery plus physiotherapy vs physiotherapy alone

#### Effects on pain

**Fig 3:**
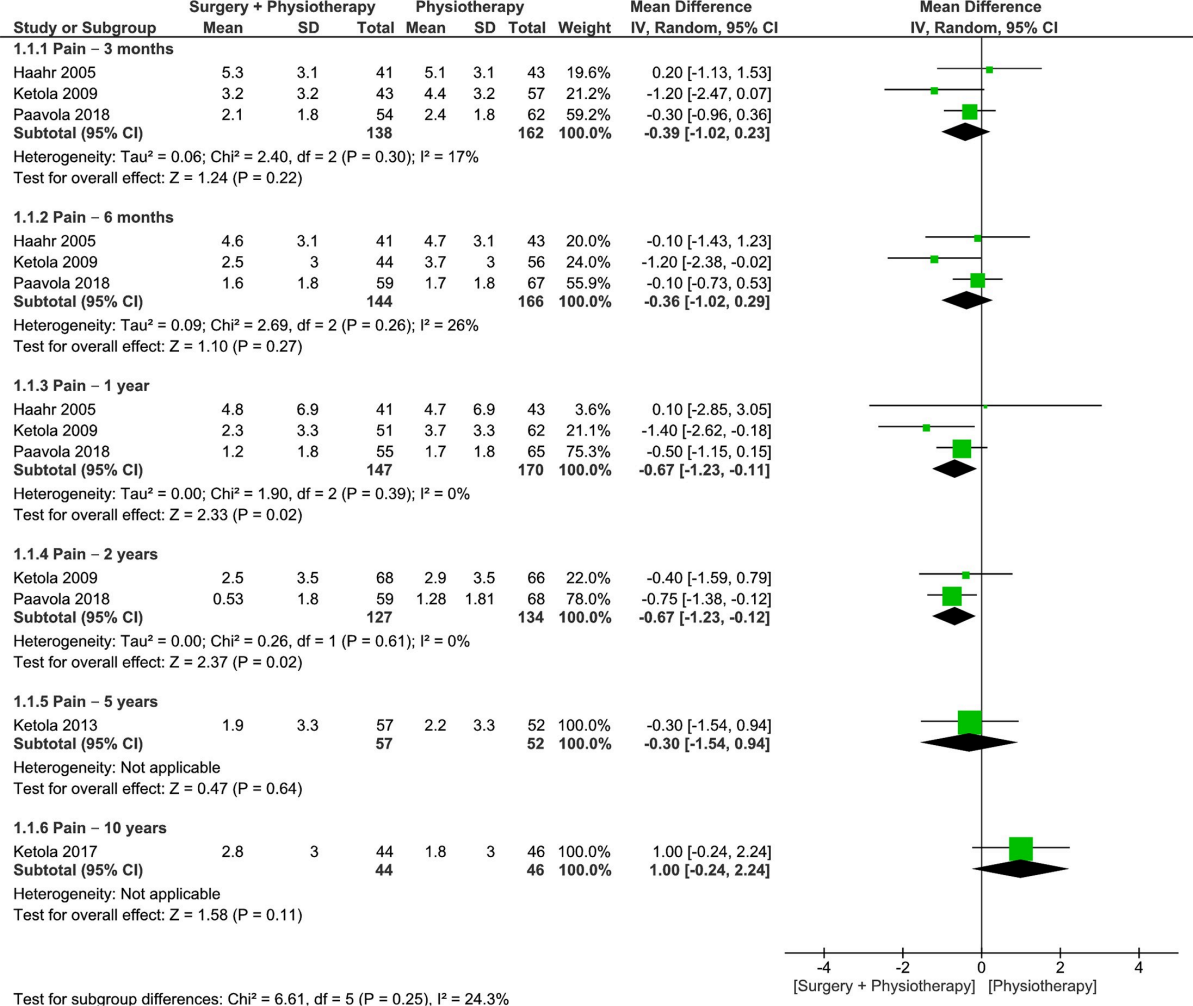
Forest plot of comparison: Surgery plus physiotherapy vs Physiotherapy alone, outcome: Pain (0–10 VAS). Lower values indicate improved pain.

Three trials were pooled to examine the effects of surgery plus physiotherapy vs physiotherapy alone on pain levels at 3- and 6-month follow ups [10,26,28]. The pooled results were not statistically or clinically different between groups at 3- and 6-month follow ups (moderate quality, 3 RCTs, 300 patients, WMD -0.39, 95% CI: -1.02 to 0.23, p = 0.22, Fig 3; moderate quality, 3 RCTs, 310 patients, WMD -0.36, 95% CI: -1.02 to 0.29, p = 0.27, Fig 3) respectively[10,26,28]. At 1-year follow up, the pooled results from 3 trials displayed statistically significant differences in favor for surgery plus physiotherapy, however there were no clinically important differences between groups (moderate quality, 3 RCTs, 317 patients, WMD -0.67, 95% CI: -1.23 to -0.11, p = 0.02, Fig 3) [10,26,28]. We found similar results in favor for surgery plus physiotherapy at 2-years follow up (moderate quality, 2 RCTs, 261 patients, WMD -0.67, 95% CI: -1.23 to -0.12, p = 0.02, Fig 3) [10, 28]. Our results from a single trial demonstrated no statistically or clinically important differences between groups at 5- and 10-years follow up (low quality, 1 RCT, 109 patients, WMD -0.30, 95% CI: -1.54 to 0.94, p = 0.64, Fig 3; low quality, 1 RCT, 90 patients, WMD -1.00, 95% CI: -0.24 to 2.24, p = 0.11, Fig 3) respectively [29,31]. Heterogeneity was absent or minimal for all analyses. Because the 95% CIs at 3-, 6-months, 1- and 2-years follow up excluded the MCID of 1.5 points on a 10-point scale [21], it is likely that physiotherapy alone is no worse than surgery plus physiotherapy in lowering pain levels. However, we are unable to make this same declaration for the results at 5- and 10-years as it remains possible that either approach could offer superior outcomes in terms of lower pain levels. More data is required to make a definitive conclusion.

#### Effects on function

**Fig 4:**
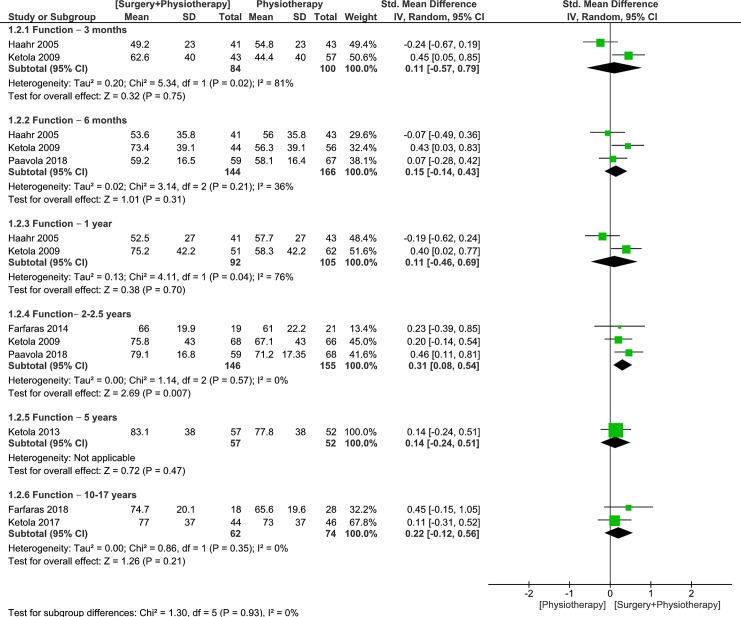
Forest plot of comparison: Surgery plus physiotherapy vs Physiotherapy alone, outcome: Function (0–100). Higher values indicate improved Function.

Up to three trials were pooled to examine the effects of surgery plus physiotherapy vs physiotherapy alone on function at 3- and 6-months, 1- and 2–2.5 years follow up [26,28,30]. The pooled results were not statistically significant between groups at 3-, 6-month and 1-year follow ups (very low quality, 2 RCTs, 184 patients, SMD 0.11, 95% CI: -0.57 to 0.79, p = 0.75, Fig 4 [26,28]; moderate quality, 3 RCTs, 310 patients, SMD 0.15, 95% CI: -0.14 to 0.43, p = 0.31, Fig 4 [26,28]; very low quality, 2 RCTs, 197 patients, SMD 0.11, 95% CI: -0.46 to 0.69, p = 0.70, Fig 4) [26,28], respectively. Confidence intervals were wide and could not rule out a clinically important effect of either treatment. At 2–2.5 years follow up [28,30], the pooled results from 3 trials displayed statistically significant differences in favor for surgery plus physiotherapy, however there were no clinically important differences between groups (low quality, 3 RCTs, 301 patients, SMD 0.31, 95% CI: 0.08 to 0.54, p = 0.007, Fig 4) [28,30]. At 5- and ≥ 10-years follow up, our results displayed no statistically or clinically important differences between groups (low quality, 1 RCT, 109 patients, SMD 0.14, 95% CI: -0.24 to 0.51, p = 0.47, Fig 4 [29]; low quality, 2 RCTs, 136 patients, SMD 0.22, 95% CI: -0.12 to 0.56, p = 0.21, Fig 4) [11,31] respectively[11,29,31].

Heterogeneity was high at 3-months and 1-year follow ups (downgraded the evidence by one level), and absent or minimal for the rest of the analyses. Because the 95% CIs at 6-months follow up excluded the MCID of 0.5 SD [22], it is likely that physiotherapy alone is no worse than surgery plus physiotherapy in improving function. At 3-months, 1-, 2–2.5, 5- and ≥ 10-years, only the upper boundary of the 95% CI indicated the possibility of a moderate effect (≥0.50) in favor of surgery plus physiotherapy. Therefore, for the majority of patients, there is definitely no clinically meaningful difference between surgery plus physiotherapy and physiotherapy alone.

### Surgery plus physiotherapy vs placebo (surgery) plus physiotherapy

#### Effects on pain

**Fig 5:**
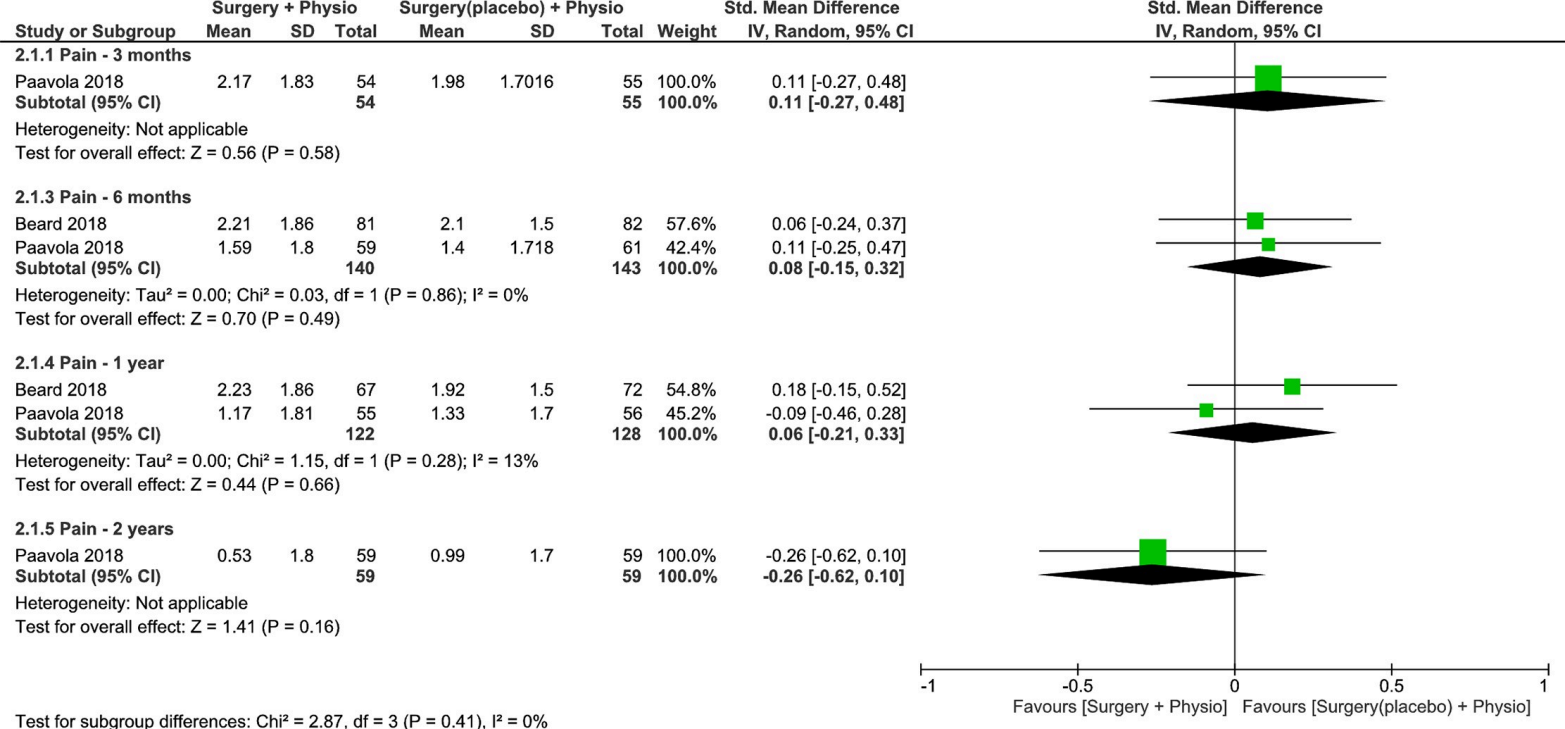
Forest plot of comparison: Surgery plus physiotherapy vs Placebo surgery plus Physiotherapy, outcome: Pain (0–10 VAS). Lower values indicate improved Pain.

Trials were pooled to examine the effects of surgery plus physiotherapy vs placebo surgery plus physiotherapy on pain levels at 3-, 6-months, 1- and 2-years follow up [9,10]. The results were not statistically or clinically different between groups at 3-, 6-months, 1- and 2-years follow up (low quality, 1 RCT, 109 patients, SMD 0.11, 95% CI: -0.27 to 0.48, p = 0.58, Fig 5 [10]; moderate quality, 2 RCTs, 283 patients, SMD 0.08, 95% CI: -0.15 to 0.32, p = 0.49, Fig 5 [9,10]; moderate quality, 2 RCTs, 250 patients, SMD 0.06, 95% CI: -0.21 to 0.33, p = 0.66, Fig 5 [9,10]; low quality, 1 RCT, 118 patients, SMD -0.26, 95% CI: -0.62 to 0.10, p = 0.16, Fig 5) [10], respectively. Heterogeneity was absent or minimal for all analyses. Because the 95% CIs at all the follow ups excluded the MCID of 1.5 points [21], on a 10-point scale or 0.5 SD (PainDETECT) [22], it is likely that placebo surgery plus physiotherapy is no worse than surgery plus physiotherapy in lowering pain levels.

#### Effects on function

**Fig 6:**
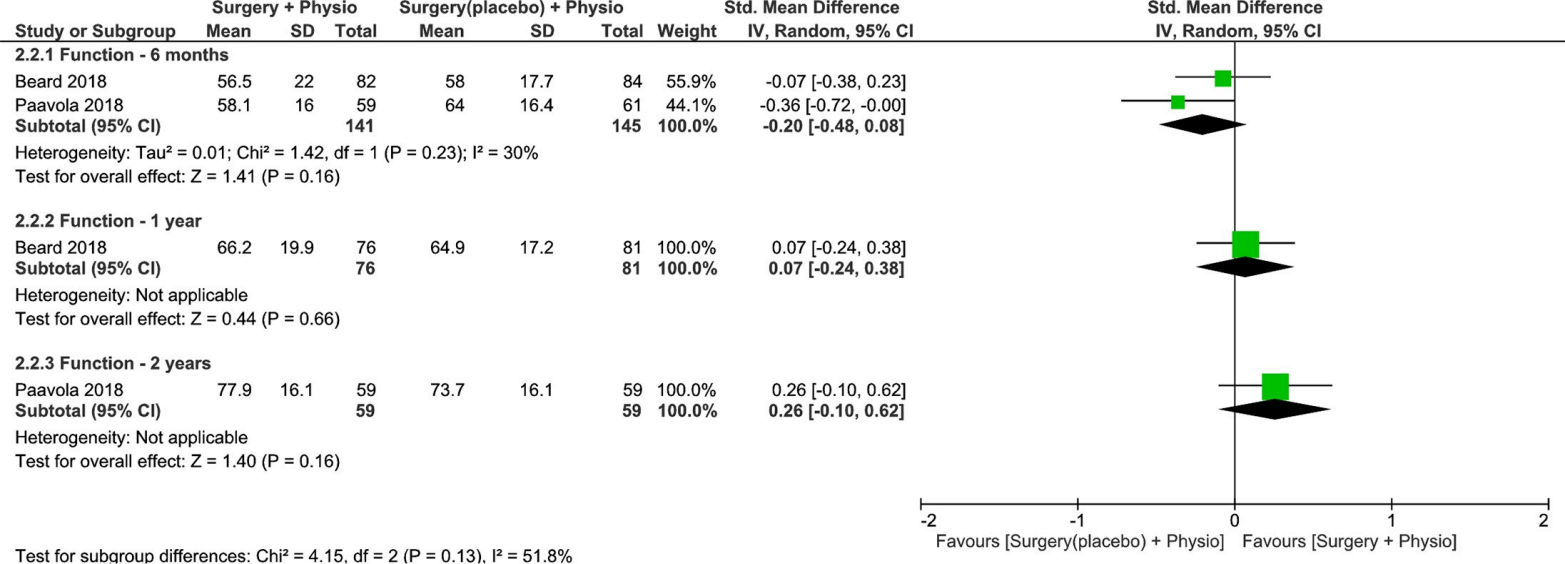
Forest plot of comparison: Surgery plus physiotherapy vs Placebo surgery plus physiotherapy, outcome: Function (0–100). Higher values indicate improved Function.

Trials were pooled to examine the effects of surgery plus physiotherapy vs placebo surgery plus physiotherapy on function at 6-months, 1- and 2-years follow up [9,10]. The results were not statistically or clinically different between groups at 6-months, 1- and 2-years follow up (low quality, 2 RCT, 286 patients, SMD -0.20, 95% CI: -0.48 to 0.08, p = 0.16, Fig 6 [9,10]; low quality, 1 RCT, 157 patients, SMD 0.07, 95% CI: -0.24 to 0.38, p = 0.66, Fig 6 [9]; low quality, 1 RCTs, 118 patients, SMD 0.26, 95% CI: -0.10 to 0.62, p = 0.16, Fig 6) [10], respectively. Heterogeneity was low for the pooled analysis. Because the 95% CIs at 6-months and 1-year follow ups excluded the MCID of 0.5 SD [22], it is likely that placebo surgery plus physiotherapy is no worse than surgery plus physiotherapy in improving function. At 2-years, only the upper boundary of the 95% CI indicated any possibility of a moderate effect (≥0.50) in favor of surgery plus physiotherapy. Therefore, for the majority of patients, there is definitely no clinically meaningful difference between surgery plus physiotherapy and placebo surgery plus physiotherapy.

## Discussions

We aimed to summarise the current evidence of the effects of surgery plus physiotherapy vs placebo (surgery) plus physiotherapy or physiotherapy alone, on clinical outcomes in patients with shoulder impingement syndrome. We found no clinically meaningful differences in pain or function at any 3-, 6-months, 1-, 2-, 5- or ≥ 10-years follow up. All 11 trials identified in this review were rated at high risk of bias. This was partially due to the fact that blinding of participants and personnel to minimize performance bias was not possible and that we did not find statistical differences between groups (16/19 analyses), suggesting that the included studies may not have been biased. Therefore, we downgraded the evidence only by one level. We found no clinical importance of surgery plus physiotherapy vs physiotherapy, or surgery over placebo surgery on clinical outcomes of pain and function. Patient goals, values and shared decision-making need to be incorporated when discussing treatment options for patients with subacromial pain syndrome.

### Quality of the evidence

The rating of very-low to moderate-quality evidence per outcome across trials was based on the judgement of serious limitations–risk of bias (19 outcomes), serious imprecision (12 outcomes) and inconsistency (2 outcomes) in all the outcomes across trials. We are moderately confident that at up to 2-years of follow up, physiotherapy alone is no worse than surgery plus physiotherapy in lowering pain levels. However, the low-quality of evidence synthesized at 5- and 10-years of follow up indicates that we have limited confidence that physiotherapy alone is no worse than surgery plus physiotherapy, and it remains possible that either approach could offer superior outcomes in terms of lowering pain levels. In regard to improvements in function, we are moderately confident that at up to 6-months of follow up, physiotherapy alone is no worse than surgery plus physiotherapy. However, at 5- and ≥ 10-years of follow up, we have limited confidence that for the majority of patients, physiotherapy alone is no worse than surgery plus physiotherapy in terms of improving function. In considering placebo surgery along with physiotherapy, we have limited confidence that at up to 2-years of follow up, placebo surgery plus physiotherapy is no worse than surgery plus physiotherapy in lowering pain levels. Similarly, we have limited confidence that for the majority of patients at up to 2-years of follow up, placebo surgery plus physiotherapy is no worse than surgery plus physiotherapy in improving function.

### Agreements / Disagreements with other reviews

The results of our review are in concordance with the findings of Saltychev (2015) and Toliopoulos (2014) reviews, and further builds on the Steuri (2017) review [1,8,32]. Saltychev (2015) concluded that, there was moderate evidence indicating surgical treatment is no more effective than active exercises on reducing pain intensity caused by shoulder impingement [1]. Toliopoulos (2014) concluded that there was low- to moderate-quality evidence to demonstrate that open acromioplasty or arthroscopic, is no more effective than exercises for the treatment of rotator cuff tendinopathy [32]. Our review further builds on Steuri (2017) review [8]. Our review provides 1) more comprehensive quantitative synthesis beyond 1-year follow up and includes six additional trials, 2) ratings of the quality of evidence according to GRADE guidelines across each outcome, and 3) an analysis of precision by evaluating the MCID thresholds with the 95% confidence intervals, therefore, able to make definitive conclusions for most of the included clinical outcomes. To highlight the precision of the pooled studies, we calculated the Optimal Information Size (OIS) for both the pain and function outcomes (S2 Appendix). For the pain outcome (VAS 0–10), we specified a two-sided test with alpha (α) error rate of 0.05, beta (β) error rate of 0.2, expected difference (δ) of 1.5 (VAS units), and a standard deviation of 3.5, which was derived by pooling the SD of the included studies. This yielded an OIS of 172 patients 86 per group). The quality of evidence for surgery plus physiotherapy vs physiotherapy alone, at 5- and 10-years was downgraded by one level because our analysis of 109 and 90 patients respectively, did not meet the criteria for our calculated OIS of 172. An OIS of 308 was calculated for the function outcome (Constant 0–100) using a two-sided alpha (α) error of 0.05, beta (β) error of 0.2, expected difference (δ) of 10, and a pooled standard deviation (31.5). Similarly, the quality of evidence for surgery plus physiotherapy vs physiotherapy alone, at 3-months, 1-, 2–2.5, 5- and ≥10-years follow up, was downgraded by one level because our analysis of 184, 197, 301, 109 and 136 patients respectively, did not meet the criteria for our calculated OIS of 308. We should also comment that our OIS calculations factor in a margin of superiority (the addition of surgery to physiotherapy) or non-inferiority (the removal of surgery as a treatment option). The margin defines the minimum amount of change required to warrant practice changes. Adding a margin increases the sample size requirements. Unfortunately, the most common method used to estimate sample size does not adjust for a margin. This is one of the reasons why the 95% confidence intervals around between-group differences (even those that are statistically significant) are often still wide (the lower and upper boundary range from between-group differences that are too small to be important to those that imply an extremely large effect sizes) and therefore can only offer indeterminate results.

### Implications for research

We have limited to moderate confidence (in the meaningfulness of differences or lack of differences between groups) in our conclusion. Future well-designed large-scale RCTs investigating the effects of surgery plus physiotherapy vs physiotherapy alone, or placebo surgery plus physiotherapy, on clinical outcomes of pain and function over the long-term (≥ 5 years of follow up) are warranted to generate high quality evidence (i.e. greater confidence) to further ensure that the true effect lies close to that of the estimate of the effect. Furthermore, utilizing outcomes to capture patients’ level of satisfaction or acceptability of symptoms, and consideration of an outcome tool that is specific to the condition–such as the Western Ontario Rotator Cuff Index (WORC), are warranted.

### Implications for practice

We synthesized very-low to moderate-quality evidence and continue to suggest that physiotherapy intervention programs (with exercise component) be used as the main and first treatment approach for treatment of patients with shoulder impingement. Ultimately, the surgical option may be considered, however, it is important to note (despite the very-low to moderate quality evidence), the lack of clinically important benefits of surgery over physiotherapy (mainly exercise). In addition, patient goals, values and shared decision-making need to be incorporated when discussing treatment options for patients with subacromial pain syndrome.

### Strengths & limitations

We used MCID thresholds of 1.5 points for pain VAS scale (0–10) for pain and a standard deviation of 0.5 points for function, and pain (other than 0–10 scale, i.e. PainDETECT) to quantify meaningful change [21–22]. A standard deviation of 0.5 points was used because multiple RCTs used various outcome measures to quantify function/pain with unknown MCID thresholds, therefore, considering this lack of reported MCID thresholds and an attempt to facilitate meta-analysis of included RCTs, a standard deviation of 0.5 points for function/pain was used based on Norman et al. (2004) proposed approach [22]. We focused on RCTs and did not included conference papers, posters, abstracts or observational studies. Therefore, there might be a source of publication bias within our search strategy. We searched for all the relevant RCTs in all major databases that met our inclusion criteria stated a priori in our protocol. Two independent reviewers were used to identify, screen, extract data, and assess the risk of bias and quality of evidence. The authors of this review were not involved in the conduct of any of the included RCTs.

## Conclusions

The effects of surgery plus physiotherapy compared to physiotherapy alone on improving pain and function are too small to be clinically important at 3-, 6-months, 1-, 2-, 5- and ≥ 10-years follow up. Similarly, surgery plus physiotherapy vs placebo (surgery) plus physiotherapy comparison demonstrated no clinically important differences in terms of improving pain or function at 3-, 6-months, 1-, 2-years follow up. The evidence suggests that physiotherapy treatment programs (with exercise component) be considered as the first treatment approach, however, patient goals, values and shared decision-making remain of paramount importance and need to be considered when discussing treatment options for patients with shoulder impingement syndrome.

## Supporting information

S1 PRISMA Checklist PRISMA 2009 checklist. (PDF)

S1 File Search strategy. (DOCX)

S1 Appendix Cochrane risk of bias assessment Tool. (DOCX)

S2 Appendix Optimal information size (OIS) for outcomes pain and function. (DOCX)

## Abbreviations

RCTs: Randomized Controlled Trials
GRADE: Grading of Recommendations Assessment, Development and Evaluation
PICO: Population, Intervention, Comparator, Outcome
PRISMA: Preferred Reporting Items for Systematic Reviews and Meta-Analyses
MCID: Minimally Clinically Important Differences
SMD: Standardized Response Difference
WMD: Weighted Mean Difference
EP: Evidence Profile
SoF: Summary of Findings
SD: Standard Deviations
VAS: Visual Analogue Scale
