# Supplementary material for: The effectiveness of surgical vs conservative interventions on pain and function in patients with shoulder impingement syndrome. A systematic review and meta-analysis
Source: PLoS One. 2019 May 29;14(5):e0216961. doi: 10.1371/journal.pone.0216961 (PMC6541263; doi:10.1371/journal.pone.0216961)
Supplement: S1 File — (DOCX) [file pone.0216961.s002.docx]

| Database | Search conditions | Result *N* |
| --- | --- | --- |
| MEDLINE | #1 shoulder impingement  #2 shoulder impingement syndrome  #3 shoulder pain  #4 subacromial pain syndrome  #5 supraspinatus  #6 painful arc syndrome  #7 painful arc  #8 subacromial pain syndrome  #9 #1 OR #2 OR #3 OR #4 OR #5 OR #6 OR #7 OR #8  #10 shoulder surgery  #11 arthroscopic  #12 subacromial  #13 decompression  #14 arthroscopic subacromial decompression  #15 open subacromial decompression  #16 acromioplasty  #17 shoulder repair  #18 debride  #19 shoulder surgical  #20 #10 OR #11 OR #12 OR #13 OR #14 OR #15 OR #16 OR #17 OR #18 OR #19  #21 conservative  #22 exercise  #23 physiotherapy  #24 physical therapy  #25 therapy  #26 rehabilitation  #27 strengthening exercises  #28 stretching exercises  #29 #21 OR #22 OR #23 OR #24 OR #25 OR #26 OR #27 OR #28  #30 randomized controlled trial  #31 #9 AND #20 AND #29 AND #30 | 264 |

| Database | Search conditions | Result *N* |
| --- | --- | --- |
| EMBASE | #1 shoulder impingement  #2 shoulder impingement syndrome  #3 shoulder pain  #4 subacromial pain syndrome  #5 supraspinatus  #6 painful arc syndrome  #7 painful arc  #8 subacromial pain syndrome  #9 #1 OR #2 OR #3 OR #4 OR #5 OR #6 OR #7 OR #8  #10 shoulder surgery  #11 arthroscopic  #12 subacromial  #13 decompression  #14 arthroscopic subacromial decompression  #15 open subacromial decompression  #16 acromioplasty  #17 shoulder repair  #18 debride  #19 shoulder surgical  #20 #10 OR #11 OR #12 OR #13 OR #14 OR #15 OR #16 OR #17 OR #18 OR #19  #21 conservative  #22 exercise  #23 physiotherapy  #24 physical therapy  #25 therapy  #26 rehabilitation  #27 strengthening exercises  #28 stretching exercises  #29 #21 OR #22 OR #23 OR #24 OR #25 OR #26 OR #27 OR #28  #30 randomized controlled trial  #31 #9 AND #20 AND #29 AND #30 | 260 |

| Database | Search conditions | Result *N* |
| --- | --- | --- |
| CINAHL | #1 shoulder impingement  #2 shoulder impingement syndrome  #3 shoulder pain  #4 subacromial pain syndrome  #5 supraspinatus  #6 painful arc syndrome  #7 painful arc  #8 subacromial pain syndrome  #9 #1 OR #2 OR #3 OR #4 OR #5 OR #6 OR #7 OR #8  #10 shoulder surgery  #11 arthroscopic  #12 subacromial  #13 decompression  #14 arthroscopic subacromial decompression  #15 open subacromial decompression  #16 acromioplasty  #17 shoulder repair  #18 debride  #19 shoulder surgical  #20 #10 OR #11 OR #12 OR #13 OR #14 OR #15 OR #16 OR #17 OR #18 OR #19  #21 conservative  #22 exercise  #23 physiotherapy  #24 physical therapy  #25 therapy  #26 rehabilitation  #27 strengthening exercises  #28 stretching exercises  #29 #21 OR #22 OR #23 OR #24 OR #25 OR #26 OR #27 OR #28  #30 randomized controlled trial  #31 #9 AND #20 AND #29 AND #30 | 23 |

| Database | Search conditions | Result *N* |
| --- | --- | --- |
| PubMed | #1 shoulder impingement  #2 shoulder impingement syndrome  #3 shoulder pain  #4 subacromial pain syndrome  #5 supraspinatus  #6 painful arc syndrome  #7 painful arc  #8 subacromial pain syndrome  #9 #1 OR #2 OR #3 OR #4 OR #5 OR #6 OR #7 OR #8  #10 shoulder surgery  #11 arthroscopic  #12 subacromial  #13 decompression  #14 arthroscopic subacromial decompression  #15 open subacromial decompression  #16 acromioplasty  #17 shoulder repair  #18 debride  #19 shoulder surgical  #20 #10 OR #11 OR #12 OR #13 OR #14 OR #15 OR #16 OR #17 OR #18 OR #19  #21 conservative  #22 exercise  #23 physiotherapy  #24 physical therapy  #25 therapy  #26 rehabilitation  #27 strengthening exercises  #28 stretching exercises  #29 #21 OR #22 OR #23 OR #24 OR #25 OR #26 OR #27 OR #28  #30 randomized controlled trial  #31 #9 AND #20 AND #29 AND #30 | 295 |
