# Supplementary material for: The effectiveness of surgical vs conservative interventions on pain and function in patients with shoulder impingement syndrome. A systematic review and meta-analysis
Source: PLoS One. 2019 May 29;14(5):e0216961. doi: 10.1371/journal.pone.0216961 (PMC6541263; doi:10.1371/journal.pone.0216961)
Supplement: S2 Appendix — (DOCX) [file pone.0216961.s004.docx]

**Optimal Information Size (OIS)**

**Outcome:** *Pain levels (0 – 10) Visual Analogue Scale.*

Alpha *α* error = 0.05;

Beta *β* error = 0.2;

N=size per group;

z_x_= the z-score/standard normal deviate for a two-sided x;

δ = a clinically acceptable margin;

S^2^= Pooled standard deviation of both comparison groups;


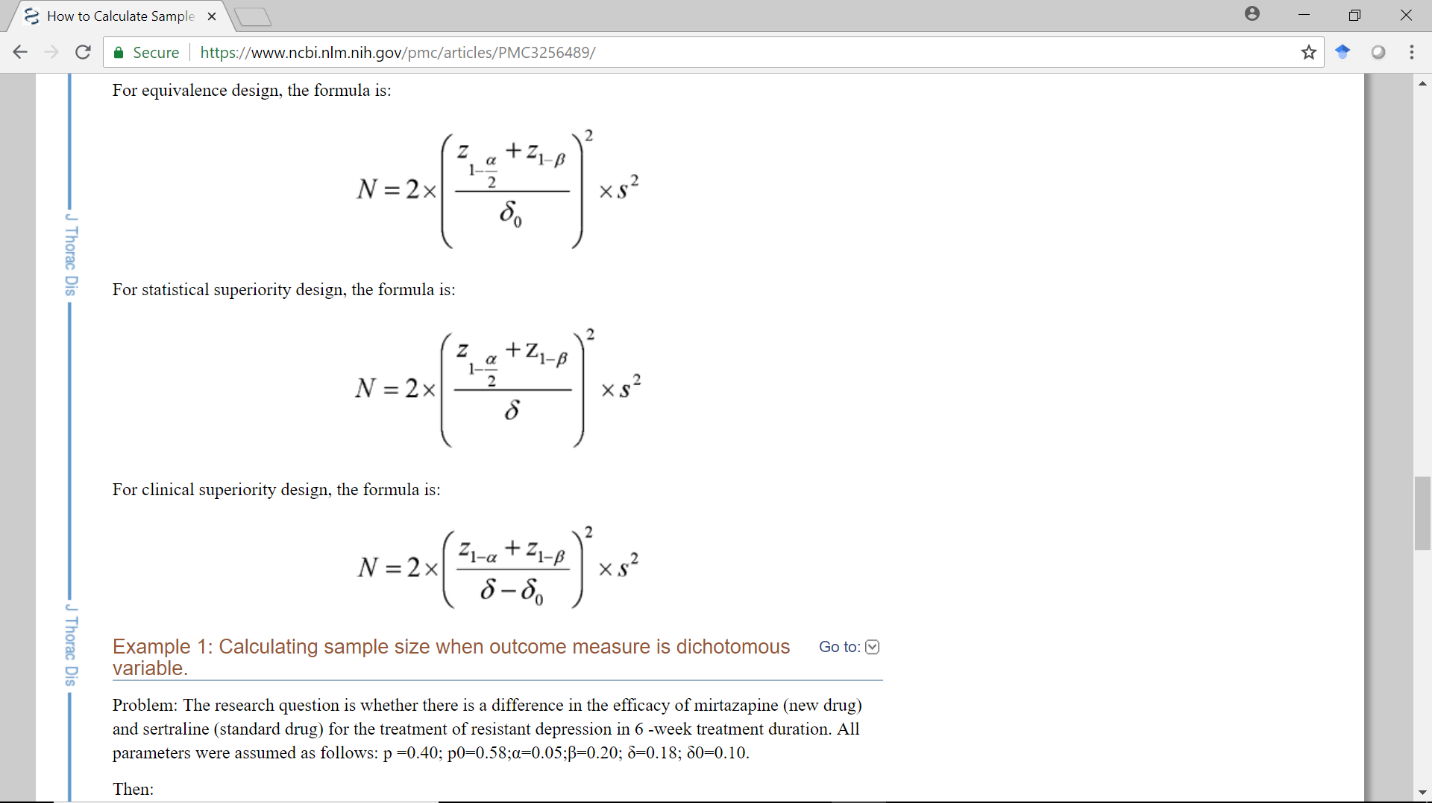


N = 2 x (1.96 + 0.842/1.5)^2^ x 3.5^2^ = 86 per group.

Total = 2 x 86 = **172**

**Optimal Information Size (OIS)**

**Outcome:** *Function (0 – 100) Constant score.*

Alpha *α* error = 0.05;

Beta *β* error = 0.2;

N=size per group;

z_x_= the z-score/standard normal deviate for a two-sided x;

δ = a clinically acceptable margin;

S^2^= Pooled standard deviation of both comparison groups;


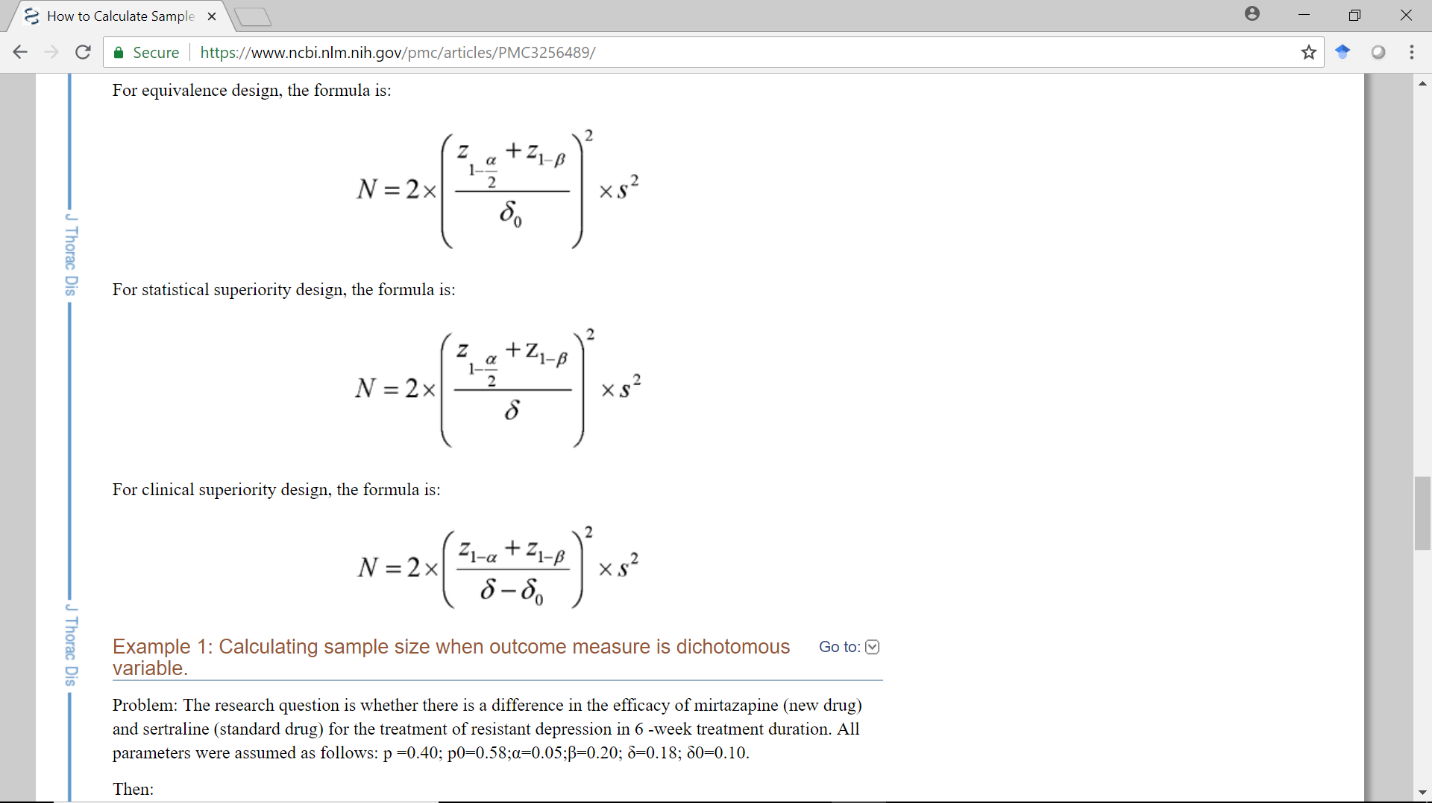


N = 2 x (1.96 + 0.842/10.0)^2^ x 31.5^2^ = 154 per group.

Total = 2 x 154 = **308**
